# Maestros of anesthesia

**DOI:** 10.7554/eLife.95064

**Published:** 2024-01-18

**Authors:** Romeesa Khan, Rodney M Ritzel

**Affiliations:** 1 MD Anderson Cancer Center UTHealth Graduate School of Biomedical Sciences Houston United States; 2 Department of Neurology, McGovern Medical School, University of Texas Houston United States

**Keywords:** microglia, anesthesia, P2Y12, calcium, Mouse

## Abstract

Microglia regulate anesthesia by altering the activity of neurons in specific regions of the brain via a purinergic receptor.

**Related research article** He Y, Liu T, He Q, Ke W, Li X, Du J, Deng S, Shu Z, Wu J, Yang B, Wang Y, Mao Y, Rao Y, Shu Y, Peng B. 2023. Microglia facilitate and stabilize the response to general anesthesia via modulating the neuronal network in a brain region-specific manner. *eLife* **12**:RP92252. doi: 10.7554/eLife.92252.

Before undergoing a surgical procedure, patients are often given a general anesthetic to put them in a state of unconsciousness. It is widely accepted that anesthetic drugs work by suppressing the activity of neurons. However, more recent studies suggest that non-neuronal cells are also involved, particularly immune cells known as microglia, which monitor the central nervous system to ensure healthy brain function is maintained. Impaired cognition following anesthesia, such as delirium and delayed recovery, have been observed in older patients. Given that microglia are known to play a role in neuroinflammation and age-related neurodegeneration, they may be key to the side effects of general anesthesia (Fan et al., 2020).

Previous studies have shown that microglia are more active and perform more surveillance in anesthetized mice (Stowell et al., 2019; Liu et al., 2019). Furthermore, differences in neuronal activity between awake and anesthetized mice correspond to shifts in both microglial function and calcium signaling (Umpierre et al., 2020). This work revealed how the signaling mechanisms that occur in microglia differ in the awake versus anesthetized state, suggesting that microglia help regulate the general anesthesia response. However, it is still poorly understood how microglia molecularly control anesthesia. Now, in eLife, Yousheng Shu (Fudan University), Bo Peng (Fudan and Nantong Universities) and colleagues – including Yang He, Taohui Liu and Quansheng He as joint first authors – report new observations showing how microglia and neurons interact in specific brain regions during general anesthesia (He et al., 2023).

First, the team investigated how depleting microglia affected the induction of and emergence from general anesthesia. To ablate the microglia, they fed mice a diet containing the compound PLX5622 which inhibits a receptor (known as CSF1R) that microglia need to survive. The PLX5622-fed mice were slower to lose the ability to flip over and stand on their feet (known as the loss of righting reflex) compared to mice given a control diet, suggesting delayed induction of anesthesia. Additionally, the mice also recovered this ability (recovery of righting reflex) faster, indicating earlier emergence from anesthesia. To establish this effect as being microglia-specific, and not due to equivalent cells outside the brain, He et al. fed mice a CSF1R inhibitor that cannot cross the blood-brain-barrier and enter the central nervous system. The rates of induction of and emergence from general anesthesia were similar to those observed in the control group, suggesting that both effects are mediated specifically by microglia. These findings were further validated by recording electrical activity in the brain and muscle, which also indicated a late induction and early emergence from anesthesia in microglia-ablated mice.

Next, He et al. looked at how microglia affect different regions of the brain during general anesthesia using the expression levels of c-Fos as a marker for neuronal activity. This revealed that microglia depletion significantly reduced neuronal activity in the parts of the brain activated by anesthesia, but increased activity in the regions activated during emergence. This region-dependent regulation was also demonstrated through a series of experiments measuring whole-cell recordings of neuronal activity. These findings build upon previous studies showing microglia to modulate neuronal activity in a region-specific manner (Badimon et al., 2020).

Finally, to better understand how microglia affect neuronal function, He et al. knocked out a microglial receptor, known as P2Y12R, that regulates neuronal crosstalk and activity when activated by purines, such as ADP (Yu et al., 2019). P2Y12R knockout was achieved in two different ways: using a selective antagonist to block ligand binding and inhibit downstream signaling, and by inactivating the gene that codes for the receptor in microglial cells through gene editing. Both methods delayed the induction of anesthesia and led to an accelerated right of recovery reflex. To validate this finding, He et al. transplanted the mice with bone marrow cells that mature into P2Y12R-negative microglia and replace the healthy microglia of the central nervous system (Xu et al., 2020). This repopulation led to similar outcomes to those observed in P2Y12R-knockout conditions.

Activation of P2Y12R enhances the level of calcium inside microglia (Jiang et al., 2017). Further experiments increasing or decreasing the amount of intracellular calcium altered how quickly the mice were induced into and emerged from an anesthetic state. These data convincingly show how disruption of purinergic receptor signaling, and consequently microglial calcium signaling, are essential for regulating anesthesia in mice (Figure 1).

**Figure 1.**
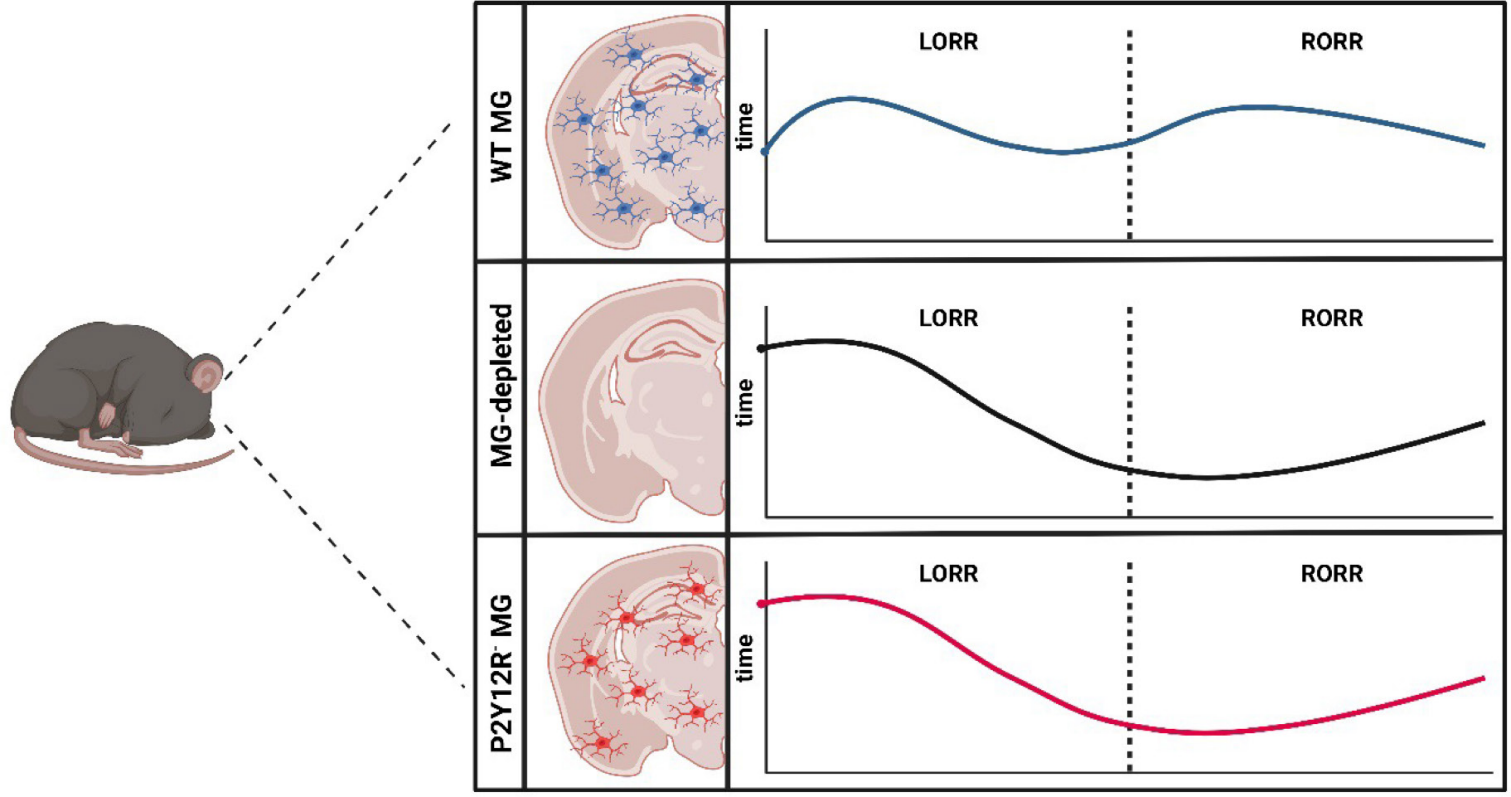
How microglia affect induction of and emergence from anesthetics. The brains of wild type mice (WT MG; top panel) are populated with immune cells known as microglia (MG; blue). Administration of general anesthetic leads to mice no longer being able to flip over and stand on their feet, known as the loss of righting reflex (LORR). This ability is then restored – known as the recovery of righting reflex (RORR) – as the mice emerge from the anesthesia (dashed line). He et al. found that depleting microglia from the brain (middle panel) caused the loss of righting reflex to occur later, while accelerating the time it took to recover the reflex. This effect was also observed when the brains of the mice were populated with microglia lacking the receptor P2Y12R (red; bottom panel), suggesting that microglia regulate the anesthetic state through this receptor.

Image credit: Figure created using Biorender.com.

The findings of He et al., alongside another recent publication which also revealed microglia regulate general anesthesia through P2Y12R (Cao et al., 2023), provide a new perspective of how immune cells regulate anesthesia. Although previously overlooked, roles for other non-neuronal cells in regulating a state of unconsciousness seem almost inevitable. Surely the vast network of neurons that are suppressed during anesthesia must significantly affect other supporting cells in the brain?

A remaining question, that is also clinically relevant, is how the elevated activity of microglia in older brains affects post-operative delirium in aging populations. A focus on microglia, which are undeniably important for maintaining brain function, may inform better practices in the clinical administration of general anesthesia. This could lead to a more comprehensive understanding of the communication between neurons and microglia within the brain.
